# Comfort During Non-invasive Ventilation

**DOI:** 10.3389/fmed.2022.874250

**Published:** 2022-03-24

**Authors:** Gianmaria Cammarota, Rachele Simonte, Edoardo De Robertis

**Affiliations:** Dipartimento di Medicina e Chirurgia, Università degli Studi di Perugia, Perugia, Italy

**Keywords:** non-invasive ventilation (NIV), acute respiratory failure (ARF), continuous positive airway pressure (CPAP), comfort, respiration

## Abstract

Non-invasive ventilation (NIV) has been shown to be effective in avoiding intubation and improving survival in patients with acute hypoxemic respiratory failure (ARF) when compared to conventional oxygen therapy. However, NIV is associated with high failure rates due, in most cases, to patient discomfort. Therefore, increasing attention has been paid to all those interventions aimed at enhancing patient's tolerance to NIV. Several practical aspects have been considered to improve patient adaptation. In particular, the choice of the interface and the ventilatory setting adopted for NIV play a key role in the success of respiratory assistance. Among the different NIV interfaces, tolerance is poorest for the nasal and oronasal masks, while helmet appears to be better tolerated, resulting in longer use and lower NIV failure rates. The choice of fixing system also significantly affects patient comfort due to pain and possible pressure ulcers related to the device. The ventilatory setting adopted for NIV is associated with varying degrees of patient comfort: patients are more comfortable with pressure-support ventilation (PSV) than controlled ventilation. Furthermore, the use of electrical activity of the diaphragm (EADi)-driven ventilation has been demonstrated to improve patient comfort when compared to PSV, while reducing neural drive and effort. If non-pharmacological remedies fail, sedation can be employed to improve patient's tolerance to NIV. Sedation facilitates ventilation, reduces anxiety, promotes sleep, and modulates physiological responses to stress. Judicious use of sedation may be an option to increase the chances of success in some patients at risk for intubation because of NIV intolerance consequent to pain, discomfort, claustrophobia, or agitation. During the Coronavirus Disease-19 (COVID-19) pandemic, NIV has been extensively employed to face off the massive request for ventilatory assistance. Prone positioning in non-intubated awake COVID-19 patients may improve oxygenation, reduce work of breathing, and, possibly, prevent intubation. Despite these advantages, maintaining prone position can be particularly challenging because poor comfort has been described as the main cause of prone position discontinuation. In conclusion, comfort is one of the major determinants of NIV success. All the strategies aimed to increase comfort during NIV should be pursued.

## Introduction

In recent years, non-invasive ventilation (NIV), including non-invasive variable positive airway pressure ventilation and continuous positive airway pressure (CPAP) (1), has progressively gained a key role in the therapy of both hypoxemic and hypercapnic acute respiratory failure (ARF) (2–6).

This has been even more true during the massive spread of severe acute respiratory syndrome-related to the novel coronavirus [severe acute respiratory syndrome coronavirus 2 (SARS-CoV-2)] pandemic, when NIV has extensively been used to cope with the massive demand for ventilatory assistance outside the intensive care unit (ICU) (7). In the management of ARF, NIV reduces the recourse to invasive mechanical ventilation (IMV), consequently avoiding the side effects related to endotracheal intubation, i.e., upper respiratory airways trauma and hemorrhage, and the use of muscle relaxants and sedatives drugs that have been demonstrated to negatively affect clinical outcomes (8).

Non-invasive ventilation has been shown to be effective in preventing intubation and improving survival of patients with ARF (9) when compared to conventional oxygen therapy (10, 11). Accordingly, NIV has been progressively employed outside the emergency department, in both clinical and surgical wards in the early treatment of ARF (12, 13).

However, this widespread diffusion of NIV has in turn allowed to find out the limits of its application. In this regard, NIV failure, defined as the need for endotracheal intubation, is the main issue while dealing with patients with NIV (14). Surprisingly, NIV is still burdened with a high failure rate (up to 40%) today, due, in most cases, to patient discomfort or rejection (15–17). During NIV, comfort is intended as the complex dynamic state based on the acceptance of non-invasive respiratory assistance in the absence of pain and emotional/physical distress (18). Accordingly, it is easy to understand why NIV is often described by patients as an extremely unpleasant experience. Patient comfort must therefore be monitored, along with vital parameters, during NIV sessions, using tools, such as the 11-point numeric rating scale (NRS) from 0 (no discomfort) to 10 (maximum discomfort) (19, 20). In keeping with a recent survey conducted in non-invasively assisted patients with the aim of assessing patients' perceptions (21), NIV is reported as a negative experience. Specifically, patients have claimed to suffer from difficult breathing, fear, and intolerance to the interface during NIV assistance. All of these factors, both combined or not, could lead to NIV failure (22). Unsuccess of NIV represents a relevant issue because it is associated to adverse clinical outcomes (23), such as mortality and prolongation of mechanical ventilation (24). Therefore, increasing attention has been progressively paid to understand all the possible factors that are responsible for poor tolerance to improve patient comfort during NIV.

In patients who underwent IMV, discomfort depends on many causes, such as pain, dyspnea, sleep deprivation, anxiety, thirst, inability to communicate, and lack of control. Among these, the management of pain and dyspnea has been demonstrated to improve clinical outcomes (25–27). A poor comfort, instead, might also be the consequence of a lack of response to NIV, suggesting the progression of the underlying disease. In keeping with previous findings (28), moderate-to-severe dyspnea after the first NIV session is associated with anxiety and is independently associated with NIV failure and subsequent intubation. In addition, the persistence of moderate-to-severe dyspnea after the first NIV session is associated with a prolonged hospital stay and mortality. Thus, the assessment of comfort overall plays a key role in the management of patients who underwent NIV. If on the one hand, discomfort depends on the NIV setting and all the strategies aimed to avoid/reduce discomfort must be pursued, on the other hand, a poor comfort is the sign of a lack of response to NIV and consequent switch to IMV is necessary.

**Figure 1:**
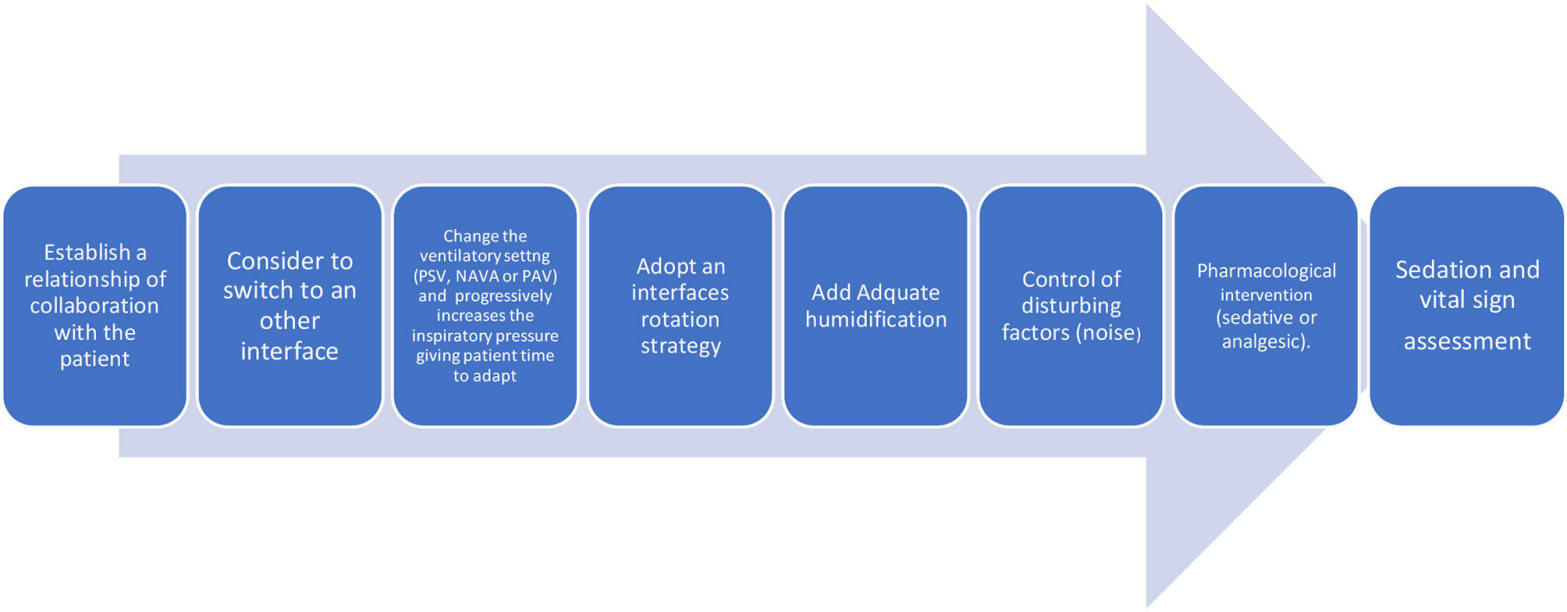
Patient intolerance bundle of intervention.

A list of possible factors responsible for poor comfort is shown in Table 1. Here are presented and discussed several causes of comfort deterioration during NIV, along with a proposal for an interventional strategy to improve patient's comfort (Figure 1).

**Table 1:** Principal causes of discomfort in non-invasive ventilation (NIV).

**Interface**
Anchor system
Ventilatory setting
Humidification
Noise
Position of the patient
Psychological distress
Anxiety
Fear
Pain

### Interface

One of the most widely investigated aspects is related to the interface dedicated to NIV. NIV interface is a potential source of pain and claustrophobia that leads to NIV discontinuation and recourse to endotracheal intubation (29). When choosing an interface, it is mandatory to take into account the time of NIV application, especially if non-invasive assistance is delivered for many hours a day (30). Specifically, it is of pivotal importance to consider the type, i.e., mask or helmet, and the size of the interface that, as much as possible, must be adapted to the patient's face and neck profile, as well the fixing system. Particularly, interface sealing system and fixing equipment play a key role in the determinism of major mask-related side effects, such as air leaks, skin breakdown, and discomfort (31).

In recent years, device manufacturers have developed different types of interfaces with various technologies and materials. There are six main classes of interfaces commercially available: the oronasal mask, the nasal mask, the full-face mask, the nasal prongs, the mouthpieces, and the helmet (32). A comparison between the characteristics of the interfaces is reported in Table 2. Many studies (33–35) have followed over time to compare the different devices and evaluate both their efficacy and tolerability in delivering NIV. A poor tolerance has been mainly reported with NIV delivered *via* the nasal mask due to the vast air loss through the mouth (36, 37); in these circumstances, the major air leaks can result in dry mouth and in less effective ventilation due to a precarious patient-ventilator interaction, in terms of wrong inspiratory triggering and cycle off (38). Nevertheless, it is worth to consider that in some cases, the nasal mask could be better tolerated than the face mask due to claustrophobia or a frequent cough (19).

**Table 2:** Comparison between interfaces^*^.

**Types**	**Advantages**	**Disadvantages**
Nasal mask	•Less claustrophobic •Easy to cough or expectorate •Easy to speak •Less risk of aspiration	•High incidence of leaks •Eye irritation •Higher resistance •Nasal irritation or damage
Nasal prongs	•Less claustrophobic •Easy to cough or expectorate •Easy to speak •Option for a rotating strategy	•High incidence of leaks •Nasal irritation
Mouth pieces	•Less claustrophobic •Little dead space •Option for a rotating strategy	•High incidence of leaks •Less effective for ARF
Oro-nasal mask	Good for ARF	•More claustrophobic •Possible air-leaks •Eye irritation
Total face mask	•Adequate for prominent facial anatomy •No pressure on nasal bridge •Low air-leaks	•More claustrophobic •Difficult to speak
Helmet	•Adequate for prominent facial anatomy •Low air-leaks •Easy to speak •No pressure on nasal bridge	•Can be claustrophobic •Noise •High gas flow required •Discomfort of axillae with armpit braces

* *Data from references 6, 12, and 20*.

The fixing system is necessary to maintain the interface in place during NIV. A proper closure of the fixing apparatus should also be pursued to prevent air leaks. A slack fixing system is the cause of both large and small air leaks that interfere with the effectiveness of the ventilatory assistance. Major leaks are accompanied by an increase in patient-ventilator asynchrony with a worsening in patient's workload (39). To compensate for leaks, the ventilator machine must deliver an increased inspiratory assistance that could result in a worse patient comfort (40). Small air leaks are accompanied by a reduced comfort also, as they can be responsible for eye irritation and produce noise (32). Conversely, an excessively tightened anchoring system can lead to pressure ulcers, with consequent NIV interruption (32). To reduce the risk of skin damage during NIV, a bundle of interventions has been proposed suggesting a rotation strategy of NIV interface application, a proper tightening of the fixing system, and the use of anti-ulcers devices, i.e., appropriate barrier tapes, cushioning, and adjustable pads between mask and face (38).

Rotating interfaces can be a useful strategy not only to avoid skin lesions but also to increase NIV tolerance, as supported by data showing a reduction in NIV failure rate when a rotational strategy of interface encompassing both mask and helmet application was adopted (41).

Problems related to air leaks and skin injuries have been partially resolved with the advent of the helmet (42). In fact, this device has been shown to have a greater tolerability over time and a lower rate of NIV interruptions when compared to masks (43, 44). In addition, the helmet allows the administration of oral nutrition and fluids along with therapy without interrupting NIV (45, 46).

In terms of effectiveness, unfortunately, the helmet is accompanied by less-efficient rates of pressurization and triggering performance when compared with the mask (47). In addition, the anchoring system is a well-recognized concern for traditional helmet (44). The armpit braces holding the helmet in place can cause discomfort and axillary skin lesions, leading to discontinuation of NIV (44). To overcome these side-effects, a new helmet equipped without armipt braces has been recently introduced (48). The new helmet also shows better performance of ventilatory assistance, particularly, in terms of ventilator machine triggering and pressurization rate (49). Due to all these advantages, a new generation of the helmet appears to be the most reasonable choice in patients who require NIV for prolonged periods, thanks to the better patient-ventilator interaction provided (50).

In selected patients and when clinical status allows, a rescue trial of high flow nasal cannula (HFNC) oxygen therapy can be tried as an alternative in case of intolerance to the various interfaces used for NIV (51). The HFNC is an open system of oxygenation therapy that can be employed to overcome the drawbacks related to the NIV interface (52, 53). According to recent findings (54) obtained in a cohort of sepsis critically ill patients weaned from IMV, the HFNC group showed a better comfort and a lower incidence of facial pressure ulcers and delirium when compared to NIV delivered *via* facial mask.

### Ventilatory Setting

The ventilatory setting adopted for NIV is associated with varying degrees of patient comfort: patients are more comfortable with pressure-support ventilation (PSV) than volume-controlled ventilation (55). However, the volume-targeted mode may be beneficial in patients with acute and marked modifications of respiratory system mechanical properties or in the case of hypercapnic encephalopathy with modifications in respiratory drive (56, 57). Volume control continuous mandatory ventilation during NIV has been employed in patients with amyotrophic lateral sclerosis (58) and volume-targeted modes of ventilation are used in patients affected by chest wall disorders (59–61) and obesity associated with chronic respiratory failure (62). During PSV, the degree of comfort shows a U-shaped trend: pressure at the extreme levels of assistance, both low and high inspiratory supports, corresponds to a reduced comfort on NIV (63). In addition to the pressure setting, the extent of patient-ventilator interaction expressed in terms of asynchrony event occurrence is also important. Indeed, asynchrony is defined as a condition in which there is a mismatch between the patient's own breathing drive and the mechanical action of the ventilator (64). According to several studies (65, 66), a high incidence of asynchronies is associated to a poor NIV tolerance.

On this basis, new ventilatory modalities aimed at increasing the degree of synchrony between patient and ventilator machine have been demonstrated to improve patient's comfort during NIV. Compared to pneumatically triggered and cycled-off PSV, the use of the electrical activity of the diaphragm (EADi) to drive the “neural”-pressure ventilation (67–69) as well as the delivery of ventilatory assistance in proportion to patient's effort (70, 71) has demonstrated to ameliorate patient-ventilatory synchrony and comfort.

The ventilator machine is obviously important during NIV. In line with recent results (72), the asynchrony events are significantly reduced with a dedicated NIV ventilator machine than with ICU ventilators equipped with an NIV algorithm, probably thank to a more effective and specific compensation system for air leaks (37). Always in terms of patient-ventilator synchrony, air leaks by promoting the dispersion of the inspiratory gas flow are the major determinants of auto-triggering events that put the patients at risk for rebreathing of exhaled gas and volotrauma (39).

### Asynchrony

Optimal patient-ventilator interaction may be of pivotal importance in NIV success. According to recent findings, high rates of asynchrony also occur during NIV. It has been demonstrated that the ability of ICU physicians to detect patient-ventilator asynchrony during NIV by inspection of flow and pressure waveforms is low. Moreover, the asynchrony detection is slightly higher with mask than with helmet and the rate of proper detection is inversely related to the prevalence of asynchrony. In patients who underwent NIV, ineffective efforts are more frequently observed with the helmet while double triggers are more recurrent with mask (73). Regarding autotriggers, no difference is reported between mask and helmet NIV. Moreover, pneumatic triggers are characterized by delays in the ventilator assistance onset and interruption, defined as inspiratory and expiratory triggers delays, respectively (49, 74).

Several strategies, such as the use of ventilators with algorithms for air-leak detection and compensation, application of leak-insensitive ventilatory modes, reduction of the applied pressure, and choice of the appropriate interface, may reduce the number of asynchronies during NIV. Moreover, the application of the neural trigger in delivering NIV has been reported to improve asynchronies, by reducing the delay from neural effort onset to inspiratory assistance initiation and reducing the incidence of ineffective efforts (49, 74).

### Humidification

Inadequate humidification during NIV assistance may cause patient distress because it is associated with upper airway mucosa dryness and nasal congestion (75). Thus, an adequate humidification must be pursued to improve respiratory comfort and prevent drying of bronchial secretions (76). Humidification can be achieved with a passive heat-moisture exchanger (HME), or through actively heated humidification, two systems that overlap in terms of major clinical outcomes, i.e., ICU stay, intubation rates, or mortality (77). It must be considered that once installed in the ventilatory circuit, HME increases the dead space and the flow resistance of the circuit with detrimental effects on patient's respiratory load (78). Furthermore, the effectiveness of the HME is compromised in the presence of air leaks (79). Active humidification during NIV may be considered for those patients who suffer from the excessive dryness of inhaled gas (38). However, when NIV is delivered through a helmet and an active humidification system is installed, attention must be paid to the increase in condensation on the inner surface of the interface, because the reduced visibility worsens the visual contact with the patient (77).

### Noise

Surrounding noise may negatively affect patient's comfort during NIV. Recently, a “bundle of interventions” has been proposed to improve the comfort in patients undergoing NIV, such as noise reduction (80). Noise exposure during NIV can be a relevant concern especially in presence of air leaks, mainly when NIV is delivered through mask (31). Minimizing the gas loss by repositioning the mask, applying a linear sealing on the face to reduce the gap between interface cushion and skin, and changing the type of mask for NIV can help to reduce the noise associated to air leaks (24). Despite the lower incidence of leakages, noise is also a significant problem when helmet NIV/CPAP is adopted due to the high gas flow system employed (31). To face off this problem, the application of earplugs, sound traps, and circuit tubes with smooth inner surfaces, as well as trying to limit, when possible, unnecessarily high flows, has been suggested as conceivable solutions (81).

### Position

The optimization of patient's position also plays a key role in assuring comfort during NIV (79). The sitting or semi-recumbent position is suggested during NIV to assure a high level of comfort to patients and a side-lying position can be obtained to remove pressure from a pendulous abdomen as in case of pregnancy or obesity (79). Recently, the use of the prone position has been introduced in patients with ARF, particularly those with Coronavirus Disease-19 (COVID-19) disease (82–84). The analysis of this rescue therapy is better explained in the last paragraph on the COVID-19 pandemic.

### Other Factors

Patient's emotional state is a major determinant of NIV success. In the case of intolerant patients, it is suggested to try a strategic relational approach. To preserve and/or improve patient's comfort and tolerance to NIV, it is fundamental to establish a trust relationship with patients, by reassuring them during ventilatory assistance, providing information on expected benefits of NIV, and involving them in the process of care (85).

### Sedation

When none of the non-pharmacological strategies listed above are successful, analgo-sedative medications schemes can be employed to manage agitation during NIV (86).

Agitation can be caused by several factors, such as fear, pain, anxiety, sleep deprivation, fever, and hypoxia (87). To face off pain affecting the musculoskeletal compartment with consequent stiffening of the chest wall and diaphragm, the administration of simple analgesics, such as acetaminophen, non-steroidal anti-inflammatory drugs, or opioid, should be considered (87).

In case of agitation due to anxiety or intolerance, the choice must fall on sedative drugs. It has been demonstrated that sedation strategy could reduce the rate of NIV failure (88). Sedation facilitates ventilation, calms anxiety, promotes sleep, and modulates the autonomic system responses to stress, such as tachycardia and hypertension, with a final improvement of patient's adaptation to NIV (89, 90). Several studies have demonstrated the efficacy and safety of sedation during NIV using dexmedetomidine, midazolam, propofol, and remifentanil (91, 92). According to the previous investigation (90), benzodiazepines (33%) and opiates (29%) are the most often selected sedative agents for NIV.

In choosing the drug, the intrinsic characteristics and clinical effects of the various pharmacological categories must be considered, mainly taking into account the effects exerted by the drug on patient's own respiratory drive. Benzodiazepines should preferentially be avoided in the elderly with agitation due to the risk of paradoxical the effect and of promoting a state of delirium (87). In addition, the benzodiazepines pharmacokinetics profile is prone to accumulation in the case of obese patients or in those subjects with renal injury or low albumin levels (93).

Propofol, thanks to its pharmacokinetic rapidity, is a particularly attractive sedative agent in NIV. However, in the choice of the propofol sedation regimen dose, it is of pivotal importance because propofol has shown to adversely affect the breathing pattern and the respiratory drive, as well as gas exchange, proportionally to the rate of its infusion (94); in this context, it has been effectively used even with a target-controlled infusion (95).

Dexmedetomidine, a selective α2 agonist with intrinsic properties of sedative and analgesic effects, may be useful for sedation of NIV patients, due to its limited effect on the respiratory pattern. According to previous findings (90) net of the sedation target, dexmedetomidine-based sedation is superior to midazolam in terms of pharmacokinetics manageability.

Remifentanil is a short-acting opioid proven to be safe and effective to achieve optimal sedation in case of intolerance to NIV (96). In keeping with a recent investigation (97), a remifentanil-based sedation plan has demonstrated the same efficacy in ameliorating moderate to severe NIV intolerance, as dexmedetomidine.

A separate description of the advantages and disadvantages of sedative drugs in NIV is summarized in Table 3.

**Table 3:** Advantages and disadvantages of sedative drugs in NIV^*^.

**Drugs**	**Advantages**	**Disadvantages**
Midazolam	•Good efficacy •Hemodynamic stability	•Increased risk of delirium and paradoxical agitation •Accumulation in critically ill patients who are obese, have low albumin levels, or renal failure
Propofol	Advantageous pharmacokinetic profile	Can cause hypotension and apnea
Dexmedetomidine	•No respiratory depression •Providing sedation, anxiolysis and analgesia •Seems superior to midazolam in terms of maintaining sedation with fewer dose adjustments	•Bradycardia and hypotension •Cautiously in patients with hemodynamic instability
Remifentanil	•Metabolism not affected by hepatic or renal dysfunction •Easy to titrate to effect •No accumulation	•Chest wall rigidity •Nausea and Vomiting

* *Data from references 75–76, 83*.

Regardless of the sedation plan adopted, sedation assessment is of pivotal importance during NIV, through subjective scales (e.g., Richmond agitation-sedation scale) or tool, i.e., bi-spectral index, entropy. The sedation assessment, at regular time intervals, allows to provide the desired target of sedation and to avoid hypersedation (66).

Regarding the concern related to the respiratory drive depression by sedative medications, it is worth to remark that sedation assessment must be assured whatever the therapeutic scheme adopted. Therefore, sedative and anxiolytic drugs should be administered in the appropriate environment, staffed with well-trained personnel in the monitoring of vital signs and sedation depth and airway emergencies management (98).

### Novel COVID-19 Pandemic

The massive spread of COVID-19 outbreak has put in crisis the surge capacity response of whole sanitary systems worldwide (99). In particular, ICU surge capacity response has been severely stressed by enormous requests for ventilatory assistance due to hypoxemic acute respiratory distress syndrome (ARDS) COVID-19 (100). To stabilize the respiratory condition and avoid intubation, NIV has been used outside the ICU (101). In this context, all the strategies finalized to increase the success of NIV have been pursued. Thus, awake prone position (APP) has been introduced as a rescue therapy in patients who underwent NIV, to ameliorate oxygenation and possibly avoid intubation (82–84).

Despite these advantages, maintaining an APP for long-lasting sessions could be very challenging. In fact, the main cause of interruption of APP has been shown to be scarce comfort (101).

The prone position reduces the compliance of the chest wall, leading to an increase in the work of breathing, and generating discomfort (7). In addition, the patients are requested to lay in an obligated position for several hours a day. According to recent data (102), when APP is employed at the expense of a comfort reduction, the consequent rise in diaphragmatic activity puts the patients at risk for IMV. Thus, to increase the chance of success of NIV combined with APP, management strategies must be implemented to increase comfort and facilitate patient's adaptation (103).

However, during the current COVID-19 pandemic, the importance of close monitoring of the patient in NIV has clearly emerged, as despite its clear benefits, a delay in intubation turns out to be associated with worse outcomes (104–106).

Patients with delayed onset of invasive ventilation have increased mortality and more severe pulmonary sequelae in terms of lung carbon monoxide diffusion capacity (DLCO) and radiological imaging (105). One possible explanation may be that maintaining patients with NIV when not appropriate can trigger patient self-induced lung injury (P-SILI) due to increased inspiratory efforts (105). Therefore, in addition to NIV comfort, it is of pivotal importance to monitor predictors of failure of NIV, i.e., no change or worsen in pH, blood gases, respiratory rate, and agitation (19), to early intervene with intubation and not worsen patients' prognosis.

## Conclusions

In conclusion, net of the underlying pathological disease, enhancing the patient comfort, seems the best strategy to improve the NIV rate of success, especially when NIV is administered for a prolonged period of time, also in combination with APP as rescue therapy. Accordingly, a strict comfort assessment with the “*ad hoc*” corrective measures is mandatory to prevent NIV discontinuation related to poor patient's tolerance.

## Author Contributions

GC and RS proposed the project and conducted the research. GC and RS wrote the manuscript while RS was responsible for tables and figure. ED revised the manuscript. All authors approved the final version of the manuscript.

## Funding

This study was financially supported by HORIZON 2020, European Commission, ENVISION–Intelligent pug-and-play digital tool for real-time surveillance of COVID-19 patients and smart decision-making in Intensive Care Units (Grant No. 101015930).

## Conflict of Interest

The authors declare that the research was conducted in the absence of any commercial or financial relationships that could be construed as a potential conflict of interest.

## Publisher's Note

All claims expressed in this article are solely those of the authors and do not necessarily represent those of their affiliated organizations, or those of the publisher, the editors and the reviewers. Any product that may be evaluated in this article, or claim that may be made by its manufacturer, is not guaranteed or endorsed by the publisher.
