# Corrigendum: Comfort during non-invasive ventilation

**DOI:** 10.3389/fmed.2023.1193466

**Published:** 2023-04-04

**Authors:** Gianmaria Cammarota, Rachele Simonte, Edoardo De Robertis

**Affiliations:** Dipartimento di Medicina e Chirurgia, Università degli Studi di Perugia, Perugia, Italy

**Keywords:** non-invasive ventilation (NIV), acute respiratory failure (ARF), continuous positive airway pressure (CPAP), comfort, respiration

In the published article, there was an error in the Funding statement. No funding statement was inserted. The correct Funding statement appears below.

The authors apologize for this error and state that this does not change the scientific conclusions of the article in any way. The original article has been updated.

